# 1,25(OH)_2_D_3_ deficiency-induced gut microbial dysbiosis degrades the colonic mucus barrier in *Cyp27b1* knockout mouse model

**DOI:** 10.1186/s13099-019-0291-z

**Published:** 2019-02-20

**Authors:** Wenjing Zhu, Jiayao Yan, Chunchun Zhi, Qianwen Zhou, Xiaoqin Yuan

**Affiliations:** 1 Department of Anatomy, Histology and Embryology, Nanjing Medical University, Xuehai Building, Rm D509, 101 Longmian Avenue, Jiangning District, Nanjing, 211166 China; 2 Department of Clinical Medicine, First Clinical Medicine College, Nanjing Medical University, Nanjing, 211166 China; 3 Key Laboratory for Aging & Disease, Nanjing Medical University, Nanjing, 211166 China; 4 Department of Pathology, Nanjing Jinling Hospital, Nanjing University School of Medicine, Nanjing, 210002 China

**Keywords:** 1,25(OH)_2_D_3_ deficiency, Inflammatory bowel disease, Gut microbiota, Colonic mucus barrier

## Abstract

**Background:**

The relationship between disturbances of the gut microbiota and 1,25(OH)_2_D_3_ deficiency has been established both in humans and animal models with a vitamin D poor diet or a lack of sun exposure. Our prior study has demonstrated that *Cyp27b1*^−*/*−^ (*Cyp27b1* knockout) mice that could not produce 1,25(OH)_2_D_3_ had significant colon inflammation phenotypes. However, whether and how 1,25(OH)_2_D_3_ deficiency due to the genetic deletion controls the gut homeostasis and modulates the composition of the gut microbiota remains to be explored.

**Results:**

1,25(OH)_2_D_3_ deficiency impair the composition of the gut microbiota and metabolite in *Cyp27b1*^−*/*−^ mice, including *Akkermansia muciniphila, Solitalea Canadensis*, *Bacteroides*-*acidifaciens*, *Bacteroides plebeius* and SCFA production. 1,25(OH)_2_D_3_ deficiency cause the thinner colonic mucus layer and increase the translocation of the bacteria to the mesenteric lymph nodes. We also found 1,25(OH)_2_D_3_ supplement significantly decreased *Akkermansia muciniphila* abundance in fecal samples of *Cyp27b1*^−*/*−^ mice.

**Conclusion:**

Deficiency in 1,25(OH)_2_D_3_ impairs the composition of gut microbiota leading to disruption of intestinal epithelial barrier homeostasis and induction of colonic inflammation. This study highlights the association between 1,25(OH)_2_D_3_ status, the gut microbiota and the colonic mucus barrier that is regarded as a primary defense against enteric pathogens.

## Background

Vitamin D is a prohormone that can be converted to the active form of 1,25-dihydroxyvitamin D3 [1,25(OH)_2_D_3_] by 1α-hydroxylase encoded by the *Cyp27b1* gene [1]. In addition to its role in regulating Ca^2+^ and Pi transport and bone mineralization, 1,25(OH)_2_D_3_ also possesses various biological activities through binding vitamin D receptor (VDR), a high-affinity nuclear receptor that transcriptionally regulates its target genes [2]. There is growing epidemiological evidence demonstrating that vitamin D-deficiency (commonly defined as serum 25(OH)D < 20 ng/ml) or vitamin D- insufficiency (serum 25(OH)D < 30 ng/ml) is related to an increased risk of inflammatory bowel disease (IBD) [3, 4]. Several studies have reported that vitamin D deficiency is often observed in patients with newly diagnosed IBD [5–7]. Conversely, high vitamin D intake can lower IBD risk [8]. In mouse models, 1,25(OH)_2_D_3_ deficiency or VDR knockout increased the risk of colitis [9–11]. In either trinitrobenzene sulphonic acid (TNBS)- or dextran sodium sulphate (DSS)-induced colitis mice models, administration of 1,25(OH)_2_D_3_ effectively reduced the disease severity [9, 12]. Therefore, vitamin D might play a protective role for IBD. The role vitamin D plays in the pathogenesis of IBD is complex and not well defined. Some investigations have shown that 1,25(OH)_2_D_3_ has a pivotal role in the development of IBD via regulating innate and adaptive immune response [13], autophagy [14] or gut barrier integrity [15]. Our prior study revealed that *Cyp27b1* disruption induced colon inflammation in mice by increasing oxidative stress and DNA damage consequently leading to induction of cell senescence and mass generation of senescence-associated secretory factors [11].

Inflammatory bowel disease, i.e. Crohn’s disease and ulcerative colitis, is chronic relapsing and incurable inflammatory conditions of the bowel with an increasing trend of incidence and prevalence [16]. IBD is one of major health problems in the Western world as about 0.5% of the general population are afflicted with this disease [17]. Although the pathogenic factors have not been clarified yet, recent studies have demonstrated that intestinal microbiota might have an essential role in the development of IBD. Many studies demonstrated a lower diversity of the microbiome in IBD patients as compared with healthy controls, but a higher abundance of certain bacterial strains such as *Enterobacteriaceae*, and a stronger mucosal adherence of the bacteria [18, 19]. Although the gut microbiota has a role in IBD pathogenesis, the exact role of dysbiosis is far from clear. Gut microbial composition could be impacted by environmental factors including diet, age or genetic factors. Some studies have shown that vitamin D influenced the function and composition of bacterial communities in the gut in a protective way against dysbiosis and experimental IBD in mouse DSS model [20]. Our prior observation indicates that *Cyp27b1*^−*/*−^ mice with 1,25(OH)_2_D_3_ deficiency displayed severe colonic inflammation at the age of 8–10 month [11]. In an attempt to examine the effect of 1,25(OH)_2_D_3_ deficiency on gut microbiota in the *Cyp27b1*^−*/*−^ mice, we used 16S rRNA sequencing to dissect the composition of the gut microbiota and found gut dysbiosis in KO mice with a thinner mucus layer. Therefore, in this study we made an hypothesis that 1,25(OH)_2_D_3_ deficiency may influence gut homeostasis and induce the enrichment of some strains of bacteria such as *A. muciniphila*, *Bacteroides*-*acidifaciens* in the *Cyp27b1*^−*/*−^ mice, thereby damaging the colonic mucus barrier that would allow a greater microbial access to the intestinal mucosa further promoting colonic inflammation.

## Results

### Induction of colonic inflammation by 1,25(OH)_2_D_3_ deficiency

**Fig. 1:**
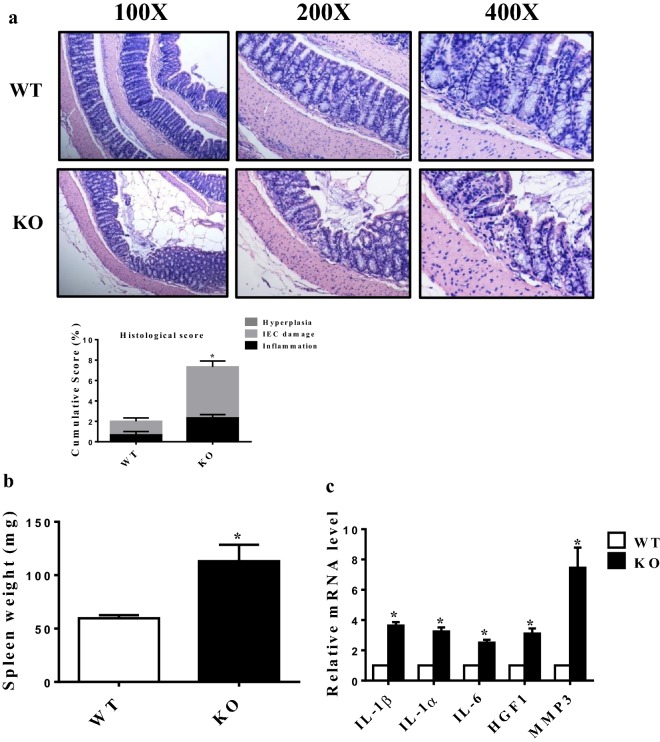
Colonic inflammation induced by 1,25(OH)_2_D_3_ deficiency. **a** Representative images and histological scores of colon sections from 8 to 10-month wild type and *Cyp27b1*^−*/*−^ mice (n = 5). Magnification, ×100, ×200, ×400. **b** Spleen weights of 8–10-month wild type and Cyp27b1^−/−^ mice. **c** Real-time RT-PCR analysis for the gene expression of IL-1ɑ, IL-1β, IL-6, HGF1, and MMP-3 on the extracts of colon from 8 to 10-month wild type and *Cyp27b1*^−*/*−^ mice. Data represent mean ± S.E.M from three independent experiments. *P < 0.05, **P < 0.01, WT: wild type, KO: *Cyp27b1*^−*/*−^

We have previously shown that *Cyp27b1*^−*/*−^ (KO) mice deficient in 1,25(OH)_2_D_3_ presented with significant colon inflammation phenotypes such as shortened colon length, disordered mucosal structure, and inflammatory cell infiltration [11]. In accord with the increased levels of inflammation at the age of 8–10 months, *Cyp27b1*^−*/*−^ mice had an increase in spleen weight and higher histological scores as compared to that of WT mice (Fig. 1a, b). *Cyp27b1*^−*/*−^ mice also had increased proinflammatory cytokines in the colonic tissue (Fig. 1c). Thus, 1,25(OH)_2_D_3_ deficiency induced colonic inflammation.

### Effects of 1,25(OH)_2_D_3_ deficiency on gut microbiota

**Fig. 2:**
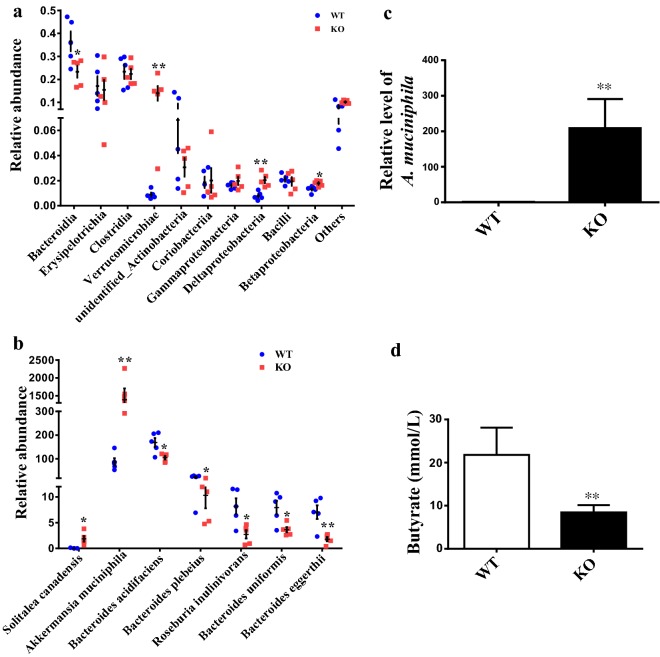
1,25(OH)_2_D_3_ deficiency alters the composition of the gut microbiota in the *Cyp27b1*^−*/*−^ mice. **a** Abundance distribution of TOP 10 bacterial taxa for 8–10-month wild type and *Cyp27b1*^−*/*−^ mice at class level. N = 5. **b** Student’s t test analysis revealed the significant changed bacteria in species including *Akkermansia muciniphila* (*A. muciniphila*), *Bacteroides*-*acidifaciens*, *Bacteroides plebeius*, *Bacteroides uniformis*, *Roseburia inulinivorans*, and *Solitalea canadensis* in *Cyp27b1*^−*/*−^ mice. **c** Relative abundance of *A. muciniphila* in feces of 8–10-month wild type and *Cyp27b1*^−*/*−^ mice. **d** Gas chromatography was performed to measure the concentration of butyrate in feces from 8 to 10-month wild type and *Cyp27b1*^−*/*−^ mice. *P < 0.05, **P < 0.01. WT: wild type, KO: *Cyp27b1*^−*/*−^

Having demonstrated that the composition of the gut microbiota was dependent on 1,25(OH)_2_D_3_, we next examined whether bacterial populations in WT mice were different from those in *Cyp27b1*^−*/*−^ mice by 16S rRNA sequencing of bacterial DNA extracted from the feces of these mice. The OTU (operational taxonomic units) data were used for obtaining taxonomic assignments of the microbiomes of the tested samples. Based on the phylum levels, *Firmicutes* and *Bacteroidetes* together represented a major part of the bacterial population in all the samples tested. Figure 2a showed the differences in the gut microbiota at the class level, in which *Bacteroidia, Erysipelotrichia, Clostridia, Verrucomicrobiae*, *Coriobacteriia*, *Gammaproteobacteria, Deltaproteobacteria*, unidentified *Actinobacteria* and *Betaproteobacteria* belonged to the top list of the most represented classes. In *Cyp27b1*^−*/*−^ mice, *Verrucomicrobiae* and *Deltaproteobacteria* were enriched, and *Bacteroidia* was decreased with significant difference in the feces. Statistical analysis revealed that *Akkermansia muciniphila* (*A. muciniphila*) and *Solitalea canadensis* were more abundant in *Cyp27b1*^−*/*−^ mice, especially *A. muciniphila* being affected to an even higher extent in *Cyp27b1*^−*/*−^ mice (Fig. 2b). Quantitative PCR analysis using primers specific to *A. muciniphila* further confirmed the increased abundance of this bacterium in *Cyp27b1*^−*/*−^ as compared to WT mice (Fig. 2c). *A. muciniphila* was a mucin-degrading bacterium, and *Solitalea canadensis* was also reported to degrade N-glycan with enzymatic secretion [21]. We also found that *Bacteroides*-*acidifaciens*, *Bacteroides plebeius*, *Bacteroides uniformis*, and *Roseburia inulinivorans*, which are assumed to yield short chain fatty acids (SCFA), were more abundant in WT mice (Fig. 2b). Butyrate, one of SCFA, is a main energy source for intestinal epithelial cells. The concentration of butyrate was decreased in the feces of *Cyp27b1*^−*/*−^ mice as determined by gas chromatography (Fig. 2d). These observations clearly indicate that 1,25(OH)_2_D_3_ deficiency could impair the composition of the gut microbiota, particularly the relative abundance of *A. muciniphila*, and SCFA-producing bacterium.

### 1,25(OH)_2_D_3_ deficiency causes degradation of the colonic mucus barrier

**Fig. 3:**
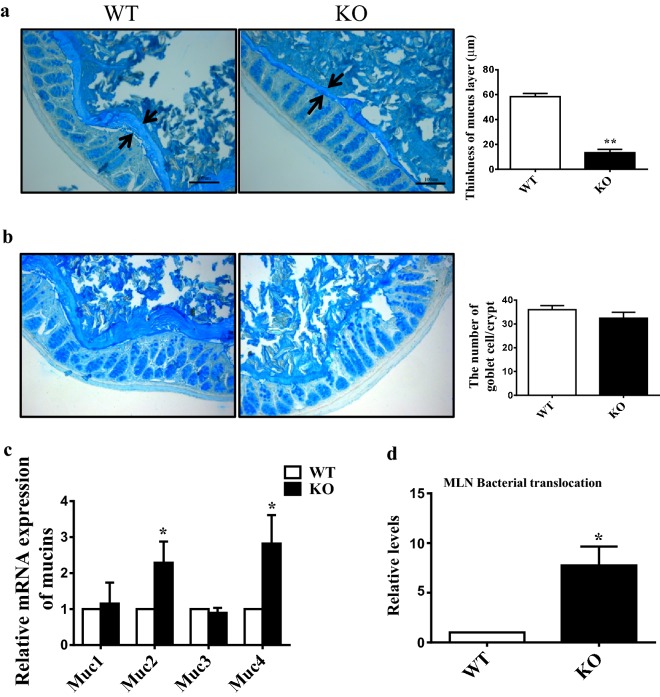
Deficiency in 1,25(OH)_2_D_3_ leads to degradation of the colonic mucus barrier. **a** Alcian blue-stained colonic sections showing the mucus layer (arrows). The histogram showing the mean percentage of the mucus layer. Scale bars, 100 μm. **b** Representative images showing Alcian blue staining of colonic crypts from 8 to 10-month wild type and *Cyp27b1*^−*/*−^ mice. The histogram showing the mean number of goblet cell density in colonic crypts. Original magnification, ×400. **c** Real-time RT-PCR analysis of the gene expression of Muc1, Muc2, Muc3 and Muc4 on extracts of colon from 8 to 10-month wild type and *Cyp27b1*^−*/*−^ mice for. **d** Relative levels of total bacteria in mesenteric lymph nodes (MLNs) from 8 to 10-month wild type and *Cyp27b1*^−*/*−^ mice. *P < 0.05, **P < 0.01. WT: wild type, KO: *Cyp27b1*^−*/*−^

Since gut microbes are important to maintain the integrity of mucus barrier, one possible mechanism by which deficiency in 1,25(OH)_2_D_3_ causes colon inflammation in *Cyp27b1*^−*/*−^ mice is acted through thinning the mucus thus predisposing mice to bacterial penetration into the intestinal mucosa. To confirm this possibility we measured the thickness of the colonic mucus layer using Alcian blue staining. We found that mucus thickness was indeed thinner in *Cyp27b1*^−*/*−^ mice relative to that in WT mice (Fig. 3a). Since the mucus layer is constantly replenished via the secretory activity of goblet cells [22] we next evaluated whether mucus production was changed in *Cyp27b1*^−*/*−^ mice. We counted the goblet cells by Alcian blue staining and quantified the expression of Muc1, Muc2, Muc3 and Muc4 by qPCR. Indeed, the number of goblet cells in *Cyp27b1*^−*/*−^ mice was lower than WT mice despite of no statistically significant differences (Fig. 3b). The same was true for the Muc1 and Muc3 mRNA expression (Fig. 3c) although the mRNA levels of Muc2 and Muc4 were higher in *Cyp27b1*^−*/*−^ mice. These results indicate that 1,25(OH)_2_D_3_ deficiency could cause a thinner gut mucus layer likely resulting from the alternation of the gut microbiota. Because thinner mucus layer could be beneficial to bacterial penetration, we then checked whether it affected the bacterial translocation to the mesenteric lymph nodes (MLNs) in *Cyp27b1*^−*/*−^ mice analyzed by qPCR method with the universal 16S rRNA primers. As shown in Fig. 3d, 1,25(OH)_2_D_3_ deficiency significantly increased the translocation of the bacteria to MLNs. These results implicate that 1,25(OH)_2_D_3_ deficiency induced the alteration of the gut microbiota to degrade the gut mucus layer leading to bacterial penetration into the gut mucosa for induction of inflammation.

### 1,25(OH)_2_D_3_ affects *A. muciniphila* colonization in gut

**Fig. 4:**
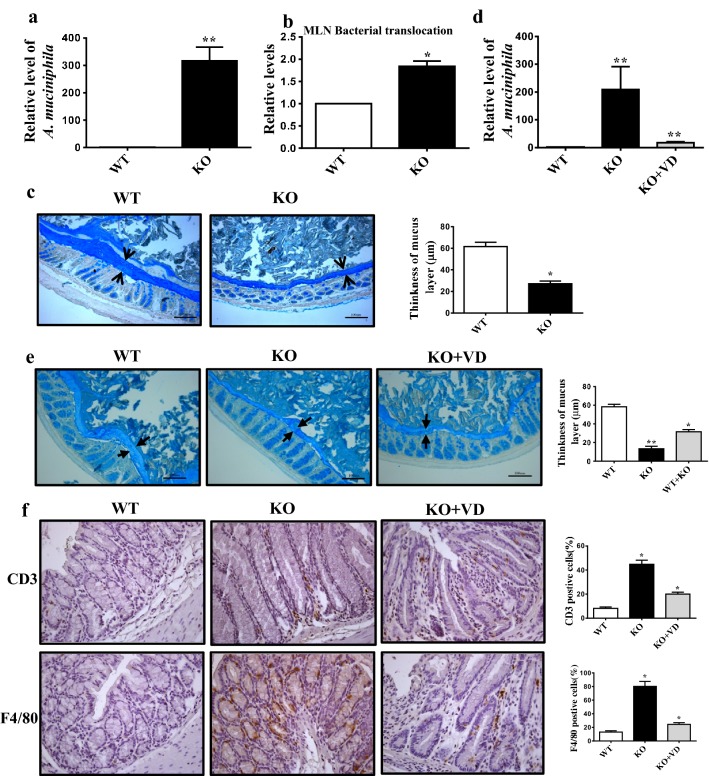
1,25(OH)_2_D_3_ affects *A. muciniphila* colonization in gut. **a** Relative abundance of *A. muciniphila* in feces and **b** Relative levels of total bacteria in MLNs from wild type and *Cyp27b1*^−*/*−^ mice with 10–12-week age. **c** The mucus layer (arrows) from wild type and *Cyp27b1*^−*/*−^ mice with the age of 10–12 weeks assessed on Alcian blue-stained colonic sections. The histogram showing the mean percentage of the mucus layer. Scale bars, 100 μm. **d** Relative abundance of *A. muciniphila* in feces from wild type, *Cyp27b1*^−*/*−^ and *Cyp27b1*^−*/*−^ mice feed with 1,25(OH)_2_D_3_ at the age of 8–10 months. **e** The mucus layer (arrows) from wild type, *Cyp27b1*^−*/*−^ and *Cyp27b1*^−*/*−^ mice feed with 1,25(OH)_2_D_3_ on Alcian blue-stained colonic sections. The histogram showing the mean percentage of the mucus layer. Scale bars, 100 μm. **f** CD3 and F4/80 expression in colon tissues from the wild type and *Cyp27b1*^−*/*−^ mice at the age of 8–10 months by immunohistochemical staining. Magnification, ×400. The histogram showing the mean percentage of the CD3 or F4/80 positive cells from five randomly selected fields. *P < 0.05, **P < 0.01. WT: wild type, KO: *Cyp27b1*^−*/*−^, KO + VD: *Cyp27b1*^−*/*−^ mice feed with 1,25(OH)_2_D_3_

Since the enrichment of *A. muciniphila* was significantly increased in *Cyp27b1*^−*/*−^ mice, we focused on the effect of 1,25(OH)_2_D_3_ deficiency on *A. muciniphila*. In order to exclude the possibility that the change of *A. muciniphila* in *Cyp27b1*^−*/*−^ mice could result from age not from gene, we checked the colon phenotype and *A. muciniphila* abundance between the young *Cyp27b1*^−*/*−^ and WT mice of 10–12 weeks. Both higher *A. muciniphila* abundance in fecal sample (Fig. 4a) and increased translocation of the bacteria to MLNs (Fig. 4b) were found in young *Cyp27b1*^−*/*−^ mice. Consistently, the thickness of colon mucus layer of *Cyp27b1*^−*/*−^ mice was thinner than that of WT (Fig. 4c) while still thicker than *Cyp27b1*^−*/*−^ mice with age of 10 months. These results suggest that 1,25(OH)_2_D_3_ deficiency increased the *A. muciniphila* abundance and this effect was more significant with the increase of age. To assess whether 1,25(OH)_2_D_3_ supplement could reduce the abundance of *A. muciniphila*, we treated *Cyp27b1*^−*/*−^ mice with 1,25(OH)_2_D_3_ supplement from weaning to 10 months. Figure 4d showed that 1,25(OH)_2_D_3_ significantly decreased *A. muciniphila* abundance in fecal samples of *Cyp27b1*^−*/*−^ mice. Likewise, *Cyp27b1*^−*/*−^ mice with 1,25(OH)_2_D_3_ supplement obviously recovered the thickness of gut mucus layer (Fig. 4e) and rescued the colon inflammation (Fig. 4f). These data suggest that 1,25(OH)_2_D_3_ could affect *A. muciniphila* colonization in gut.

## Discussion

A large body of evidences have established a strong link of low-level vitamin D to high risk of colon cancer and colonic inflammatory disease. Epidemiologic studies have shown that decreased vitamin D levels may influence the onset of IBD [8], increase clinical disease activity [7, 23, 24] and have a higher risk of malignant transformation [25, 26]. We and others also documented that in mice models, 1,25(OH)_2_D_3_ deficiency or VDR knockout was correlated with an increased risk of colitis and 1,25(OH)_2_D_3_ supplement ameliorated DSS-induced colitis [3, 9–11]. In the present study, 1,25(OH)_2_D_3_ supplement was able to rescue the inflammation occurred in *Cyp27b1*^−*/*−^ mice (Fig. 4f). While the underlying mechanism is still unclear, accumulating evidences indicate that vitamin D play a preventive role in IBD development via regulating immune response, modulating the release of inflammatory cytokines [27, 28], improving intestinal epithelial barrier function by increasing the expression of some tight junction proteins such as Occludin, Zo-1, Zo-2, Vinculin and Claudins [29, 30], inducing colon cells senescence to secret senescence-associated inflammatory cytokines [11], and increasing antimicrobial peptide synthesis and secretion [31]. Metagenomic studies have shown that vitamin D deficient diet or VDR knockout could impact the gut microbiome [20, 32].

Inflammatory bowel disease has been associated with dysbiotic microbiota due to a balance switch between commensal and pathogenic microorganisms [33–35]. For instance, the phylum *Firmicutes* is often less colonies in the feces of patients with Crohn’s disease [35, 36] whereas members of the Proteobacteria phylum such as *Escherichia coli* are commonly more abundant in patients with IBD as compared with healthy subjects [36, 37]. Bowdish and his colleagues found that alterations in age-related microbiota influenced intestinal permeability, caused age-associated inflammation, and decreased macrophage function [38]. Microbiome genome-wide association studies have discovered that defects in many human genes involving IBD are associated with an aberrant composition of the gut microbiome [39]. For example, knockout of Nod2 in mice predisposed them to colitis with lower levels of antimicrobial defensins and a higher bacterial load as compared with the control mice [40]. In the present study, we compared the microbiome composition between 1,25(OH)_2_D_3_ deficient *Cyp27b1*^−*/*−^ mice and WT mice via 16S rRNA sequencing. Our results demonstrated that the microbiomes established in WT and *Cyp27b1*^−*/*−^ were distinct (Fig. 2a), suggesting that 1,25(OH)_2_D_3_ did modulate the composition of the gut microbiota. While these associations are well fit with the roles of the gut microbiota in IBD pathogenesis, the exact mechanism underlying dysbiosis remains to be fully elucidated.

A mucus layer in the gut tract is generally considered as a protective barrier against pathogenic micro-organisms and various chemical, enzymic or physical damage. Mucus produced by goblet cells is a viscous gel that mainly consists of high-molecular-mass glycoproteins, named as mucins [41]. During evolution some mucolytic bacterial species may gain the capacity of utilizing this nutrient source [42]. Therefore, the integrity of the mucus layer is leveraged between degradation by gut bacteria and replenishment by goblet cells. The Gram-negative *A. muciniphila* is a strictly anaerobic bacterium and abundant in the human gut with the capability of degrading mucin [41]. Seregin and his colleagues found that NLRP6, which is a member of Nod-like receptor (NLR) family and are involved in the formation of inflammasomes [43], its deficiency can increase the susceptibility to DSS-induced colitis [44] and induced the enrichment of *Akkermansia muciniphila* that could function as a pathobiont by promoting colitis in a genetically-susceptible host [45]. In contrast, Lemire et al. [46] and Mamantopoulos et al. [47] found that NLRP6 did not significantly influence the intestinal microbiota at homeostasis. These differences may be resulted from several factors including the mouse lineages (NLRP6 conditional knock-out versus NLRP6 conventional knock-out) and location of mouse facilities. 1,25(OH)_2_D_3_ has been reported to be involved in the inflammasome [48], whether 1,25(OH)_2_D_3_ has a function on NLRP6 is worthy of further investigation. It has also been reported that fiber-free dietary promoted enrichment of mucus-degrading bacteria including *A. muciniphila* and *B. caccae* [49]. Consistently, our data showed that *A. muciniphila* was significantly enriched in *Cyp27b1*^−*/*−^ mice as compared to WT mice (Fig. 2b, c), and supplement of 1,25(OH)_2_D_3_ could reduce its enrichment (Fig. 4d). This indicated that 1,25(OH)_2_D_3_ could limit the colonization of *A. muciniphila*. However, vitamin D deficient high fat diet has been shown to decrease the abundance of *A. muciniphila* in ileum [21]. Such discrepancy might be due to the different mouse model and location site of *A. muciniphila*. In our study, *Cyp27b1*^−*/*−^ mice showed the long-time status of 1,25(OH)_2_D_3_ deficiency while 1,25(OH)_2_D_3_ deficient diet indicated the short-time 1,25(OH)_2_D_3_ deficiency, which might result in the different effects on gut microbiota. The role of *A. muciniphila* in colitis is not very clear. Some studies showed that it could promote colitis. For example, one study found that occurrence of colitis was substantially increased in SPF IL10^−/−^ mice administered with repeated oral gavage of *A. muciniphila* [45]. In the presence of *A. muciniphila*, Salmonella-induced colitis was worsen and ulcerative colitis patients was accompanied by active pouchitis and the IBD patients presented with treatment failure [50–52]. The mechanism underlying *A. muciniphila*-promoted colitis might be due to the degradation of the mucus layer that allows a greater microbial access to the gut mucosa. However, some studies showed that colitis was associated with a reduction in *Akkermansia muciniphila* in IBD patients [53, 54]. Therefore, a large scale of studies is needed to confirm the clinical relation of colitis and *A. muciniphila*. In fact, we found a thinner mucus layer in *Cyp27b1*^−*/*−^ mice with alterations in bacterial species such as higher amount of *A. muciniphila* (Fig. 3a) and an increase of total bacterial translocation (Fig. 1c) leading to the inflammation (Fig. 1d). Our results also showed no significant changes in the number of goblets and the compositions of mucins such as Muc1 and Muc3 between WT and *Cyp27b1*^−*/*−^ mice (Fig. 3b, c). Since the proliferation of goblet cells and the expression of mucin genes were not significantly altered, it is reasonable to conceive that thinner mucus layer in *Cyp27b1*^−*/*−^ mice may result from faster degradation of mucus layer due to the enrichment of mucin-degraded A. muciniphila in the gut rather than a reduction of mucin production itself. We further found that 1,25(OH)_2_D_3_ supplement reversed the amount of *A. muciniphila*, recovered the mucus layer and relieved the colonic inflammation (Fig. 4d–f). These findings indicate that 1,25(OH)_2_D_3_ could limit the colonization of *A. muciniphila* in gut. We and others have shown that 1,25(OH)_2_D_3_ is an important regulator of immune systems that could elicit Th2 immune responses and decrease pro-inflammatory cytokines such as IL-1, IL-6, IL-8, IFNγ and TNFα [11]. 1,25(OH)_2_D_3_ could also increase Tregs, downregulate T cell-driven IgG production, inhibit DC differentiation, and enhance protective innate immune responses [55]. Moreover, it has also been reported that 1,25(OH)_2_D_3_ promotes the production of anti-microbial peptides (AMPs), including β-defensins and cathelicidin [56, 57]. Although the mechanism was unclear, we speculated that 1,25(OH)_2_D_3_-reduced colonization of *A. muciniphila* in gut might result from activation of immune response by 1,25(OH)_2_D_3_ or antimicrobial peptide induced by 1,25(OH)_2_D_3_. In order to exclude the influence of age on *A. muciniphila*, we checked the colon phenotype and *A. muciniphila* abundance between the young *Cyp27b1*^−*/*−^ and WT mice of 10–12 weeks. Our data demonstrated that even in young mice, 1,25(OH)_2_D_3_ deficiency led to a higher *A. muciniphila* abundance in fecal sample (Fig. 4a) and increased the translocation of bacterial to MLNs and thinner mucus layer in *Cyp27b1*^−*/*−^ mice (Fig. 4b, c). It may be noteworthy that the inflammation was not significant in *Cyp27b1*^−*/*−^ mice (data not shown), which was in concert with our prior study showing that 1,25(OH)_2_D_3_ deficiency could induce colon inflammation with aging [11]. Our present study suggests that 1,25(OH)_2_D_3_ deficiency-induced higher *A. muciniphila* location in gut was gene associated but not age-related.

## Conclusions

The present study demonstrated that 1,25(OH)_2_D_3_ deficiency impacted gut homeostasis including an increased enrichment of *A. muciniphila* in *Cyp27b1*^−*/*−^ mice that might degrade the mucus layer thus allowing a greater microbial access to the intestinal mucosa and promoting colonic inflammation. The effect of 1,25(OH)_2_D_3_ on limiting the colonization of *A. muciniphila* was genetic-associated but not age-associated. Thus, the observations obtained from this study may disclose a potential new mechanism of how 1,25(OH)_2_D_3_ protects against colitis.

## Methods

### Mice

Generation of *Cyp27b1*^−*/*−^ (KO) mice and the confirmation of their genotypes were described previously [11]. Wild-type (WT) littermates served as the controls. Animals were maintained under pathogen-free conditions on a 12-h light/12-h dark cycle. 10–12 weeks or 8–10 months of male *Cyp27b1*^−*/*−^ and WT littermates were used in this study. After weaning, they were fed with rescue diet (TD96348 Teklad, Madison, WI) formulated with 1.25% phosphorus, 2% calcium and 20% lactose or injected subcutaneously with 1,25(OH)_2_D_3_ at the dose of 1 μg/kg (KO + VD) until 10–12 weeks or 8–10 months old. It was confirmed that in the *Cyp27b1*^−*/*−^ mice serum phosphorus and calcium levels were normalized and the littermates fed with the rescue diet [11].

### Assessment of colon inflammation

After euthanasia, full length of colon was taken out and washed in PBS to remove fecal matter and then opened longitudinally, and jelly-rolled for formalin fixation and paraffin embedding. Histological assessment of H&E sections was performed in a blinded fashion by a pathologist using a scoring system as previously described [58]. Briefly, each 100 × microscopic field along the length of the colon was scored separately for the presence and severity of inflammatory cell infiltration, hyperplasia, or epithelial damage. A weighted average percent for each lesion was calculated by the equation: [(1 × # of fields with score = 1) + (2 × # of fields with score = 2) + (3 × # of fields with score = 3)]/3 × total # of fields. Colon excluding the cecum was weighed after removal of feces normalized by its length (cm).

### Extraction of bacterial DNA and 16S rRNA sequence analyses

DNA was extracted from fecal samples and 16S rRNA analysis was performed. Total genomic DNA from feces was isolated by CTAB/SDS method. Amplification of the V4 region of the 16S rRNA gene was performed by PCR with Phusion^®^ High-Fidelity PCR Master Mix (New England Biolabs, Ipswich, MA) using custom barcoded primers (16S V4:515F-806R). Sequencing libraries were constructed according to the manufacturer’s recommendations on TruSeq^®^ DNA PCR-Free Sample Preparation Kit (Illumina, San Diego, CA) and index codes were added. The library quality was evaluated on the Qubit@ 2.0 Fluorometer (Thermo Scientific, Waltham, MA) and Agilent Bioanalyzer 2100 system. Finally, an Illumina HiSeq 2500 platform was used to sequence the library with 250 bp paired-end reads generated. Analysis of sequences was done through Uparse software (Uparse v7.0.1001,http://drive5.com/uparse/) [59]. Sequences with the similarity greater than 97% were assigned to the same OTUs and representative sequence for each OTU was screened for further annotation. The GreenGene Database (http://greengenes.lbl.gov/cgi-bin/nph-index.cgi) [60] was employed for analysis of each representative sequence based on RDP classifier (Version2.2, http://sourceforge.net/projects/rdp-classifier/) algorithm to annotate taxonomic information. A standard of sequence number corresponding to the sample with the least sequences was used to normalize OTUs abundance information. Based on this output normalized data, subsequent analyses of both alpha and beta diversity were performed.

### Real-time RT-PCR

Bacterial DNA was extracted from fecal samples and its concentration was measured by Nanodrop (Thermo Scientific, Waltham, MA). Total 20 ng DNA was input for qPCR using the SYBR Green reagents (Takara Bio, Shiga, Japan) on an ABI 7300 sequence detector (Applied Biosystems, Foster City, CA). Relative abundance of *A. muciniphila* in stool samples was normalized to the universal 16S rRNA gene EUB primers. Primer sequences are as follows: EUB-F: 5′-AGAGTTTGATCCTGGCTC-3′, EUB-R: 5′-TGCTGCCTCCCGTAGGAGT-3′; *A. muciniphila*-F: 5′-AGAGGTCTCAAGCGTTGTTCGGAA-3′ *A. muciniphila*-R: 5′-TTTCGCTCCCCTGG CCTTCGTGC-3′.

Total RNA was extracted from colon tissues with TRIzol reagent (Invitrogen, Grand Island, NY, USA) with the manufacturer’s protocol. 1 μg of RNA was reverse transcribed with the PrimeScript™ 1st Strand cDNA Synthesis Kit (Takara Bio, Shiga, Japan) according to the user’s manual. cDNA was used for real-time PCR analysis with gene-specific primers to determine the relative expression of genes of interest using SYBR green reagents (Takara Bio) in an ABI 7300 sequence detector (Applied Biosystems, Foster City, CA, USA). The forward and reverse primers used are listed as follows: 5′-TGGATTTGGACGCATTGGTC-3′ and 5′-TTTGCACTGGTACGTGTTGAT-3′ for GAPDH; 5′-CGGGAGGAGACGACTCTAAAT-3′ and 5′-CACGAACAGTTGTGAATCTGAGA-3′ for IL-1ɑ; 5′-GAAATGCCACCTTTTGACAGTG-3′ and 5′-CTGGATGCTCTCATCAGGACA-3′ for IL-1β; 5′-CTGCAAGAGACTTCCATCCAG-3′ and 5′-AGTGGTATAGACAGGTCTGTTGG-3′ for IL-6; 5′-ATGTGGGGGACCAAACTTCTG-3′ and 5′-GGATGGCGACATGAAGCAG-3′ for HGF1-F; 5′-TTAAAGACAGGCACTTTTGGCG-3′ and 5′-CCCTCGTATAGCCCAGAACT-3′ for MMP3. The respective forward and reverse primers were used to detect the relative expression levels of the target genes as fold changes by the 2^−△△ct^ method. The relative amount of target mRNA was normalized to GAPDH.

### Assessment of bacterial translocation

Total DNA was isolated from mesenteric lymph nodes (MLNs) and the bacterial load was measured using qPCR analysis of the universal 16S rRNA gene EUB primers in 20 ng DNA.

### Alcian blue staining, goblet cell count and mucus thickness

The tissue of colon for Alcian blue staining was fixed in Carnoy’s fixative solution (dry methanol: chloroform: glacial acetic in the ratio of 60:30:10) and embedded in paraffin following standard procedure and the paraffin-embedded tissues were then cut 5 μm thick for staining. Alcian blue staining was performed with Kit from Nanjing Jiancheng Bioengineering Institute in China in compliance with the manufacturer’s instructions. On each slide, 10 high-power fields (200× and 400× magnification) were selected randomly. Mucus layer thickness was measured according to the method previously described [61]. Goblet cells were counted and averaged over five high power fields at 400× magnification.

### Determination of butyrate/SCFA concentrations

Gas chromatography was used to analyze the lyophilized fecal samples. One gram of lyophilisate was dissolved in 5–10 volume of ddH_2_O and 1 ml supernatant was added to 0.2 ml crotonic acid/metaphosphoric acid and then centrifuged for 10 min at 12,000 rpm. Butyrate concentration in the supernatant was measured by using a GC-14B gas chromatograph (Shimadzu Deutschland GmbH, Duisburg, Germany) equipped with a flame ionization detection with a NUKOLTM capillary column (Supelco) 30 m × 0.32 mm × 0.25 μm. A combined standard solution containing acetic acid, propionic acid, isobutyric acid, butyrate, isovaleric acid, valeric acid and crotonic acid to identify the presence of butyrate. Butyrate concentration was determined by the formula: Butyrate (mM) = (Sample PA × Standard crotonic acid PA × Concentration of standard butyrate)/(Sample crotonic acid PA × Standard PA). PA: peak area.

### Statistical analysis

Data are expressed as mean ± SEM. Statistical analyses were performed by SPSS 20.0 (Abbott Laboratories, Chicago, IL). ANOVA was employed to compare the difference between WT, KO and KO + VD.
